# Functional regulation of an outer retina hyporeflective band on optical coherence tomography images

**DOI:** 10.1038/s41598-021-89599-1

**Published:** 2021-05-13

**Authors:** Shasha Gao, Yichao Li, David Bissig, Ethan D. Cohen, Robert H. Podolsky, Karen Lins Childers, Gregory Vernon, Sonia Chen, Bruce A. Berkowitz, Haohua Qian

**Affiliations:** 1 Department of Ophthalmology, the First Affiliated Hospital, Zhengzhou University, Zhengzhou, China; 2 Visual Function Core, National Eye Institute, National Institutes of Health, Bethesda, MD 20892 USA; 3 Department of Neurology, University of California Davis, Sacramento, CA USA; 4 Division of Biomedical Physics, Office of Science and Engineering Labs, Center for Devices and Radiological Health, Food and Drug Administration, Silver Spring, MD USA; 5 Beaumont Research Institute, Beaumont Health, Royal Oak, MI 48073 USA; 6 Department of Ophthalmology, Visual and Anatomical Sciences, Wayne State University School of Medicine, Detroit, MI USA

**Keywords:** Optical imaging, Retina

## Abstract

Human and animal retinal optical coherence tomography (OCT) images show a hyporeflective band (HB) between the photoreceptor tip and retinal pigment epithelium layers whose mechanisms are unclear. In mice, HB magnitude and the external limiting membrane-retinal pigment epithelium (ELM-RPE) thickness appear to be dependent on light exposure, which is known to alter photoreceptor mitochondria respiration. Here, we test the hypothesis that these two OCT biomarkers are linked to metabolic activity of the retina. Acetazolamide, which acidifies the subretinal space, had no significant impact on HB magnitude but produced ELM-RPE thinning. Mitochondrial stimulation with 2,4-dinitrophenol reduced both HB magnitude and ELM-RPE thickness in parallel, and also reduced F-actin expression in the same retinal region, but without altering ERG responses. For mice strains with relatively lower (C57BL/6J) or higher (129S6/ev) rod mitochondrial efficacy, light-induced changes in HB magnitude and ELM-RPE thickness were correlated. Humans, analyzed from published data captured with a different protocol, showed a similar light–dark change pattern in HB magnitude as in the mice. Our results indicate that mitochondrial respiration underlies changes in HB magnitude upstream of the pH-sensitive ELM-RPE thickness response. These two distinct OCT biomarkers could be useful indices for non-invasively evaluating photoreceptor mitochondrial metabolic activity.

## Introduction

Optical Coherence Tomography (OCT) is a powerful imaging tool widely used both in eye clinics and in vision research laboratories^1,2^. In addition to the more common measurements of retina laminae structure and thickness used in these settings, functional changes during light stimulation have also been noted^3–9^. For example, light–dark changes in the subretinal space (SRS) volume surrounding rod cells in vivo, observed by microelectrodes/extracellular tracer, diffusion MRI and electrophysiological recordings^10–13^, can be also be detected using commercially available OCT^3,14,15^. In the dark, mitochondria activity is increased to maintain the photoreceptor dark current^16–18^. This increased metabolism produces large amounts of waste water and CO_2_ as byproducts and acidifies the SRS^19,20^. In turn, acidification triggers RPE to pump out water from the SRS and reduces the thickness of the external limiting membrane to retinal pigment epithelium (ELM-RPE)^21–23^. This ELM-RPE change operates on a slow time scale and differs from the faster changes in OCT intensity and optical path elicited by light within a few seconds as measured by optoretinography (ORG)^8,24^. It also appears that a different mechanism is at play at the start of the light responses measured in ORG studies^25–28^.

The ELM-RPE thickness signal cascade outlined above is further supported by recent evidence. For example, reduced ELM-RPE thickness can be produced in light adapted C57BL6J mice by increasing mitochondria activity with a low dose of protonophore (i.e., uncoupler) or increasing circulating current with a phosphodiesterase 6 inhibitor^29,30^. In addition, a relatively smaller light–dark ELM-RPE thickness is found in wildtype 129S6/ev mice vs. C57BL/6J mice. 129S6/ev mice have a relatively more efficient rod photoreceptor mitochondrial respiration compared to C57BL/6J mice, which have a spontaneous loss of nicotinamide nucleotide transhydrogenase (NNT) on the mitochondrial inner membrane^15,30,31^. NNT catalyzes the reduction of NADP^+^ to NADPH supporting the detoxification of reactive oxygen species in the mitochondria and promoting efficient ATP synthesis^15,31^. In light-adapted retina of wildtype 129S6/ev mice, greater rod photoreceptor mitochondrial efficacy means a lower base level of oxygen consumption and relatively lower production of waste water than in C57BL/6J mice.

Recent studies have raised the possibility that another region within the outer retina may also show functional changes. Specifically, a hyporeflective band (HB) between photoreceptor outer segment tips and apical RPE becomes clearly visible on light-adapted human and C57BL/6J mouse OCT images^3,7,32–34^; this region is unnamed by the IN·OCT consortium^2^. However, the light–dark change of HB has not been the subject of investigations in animal or humans. In our previous studies, we noted that the HB magnitude is substantially decreased after dark-adaptation in mice^3,14,15^, but its connection with changes in ELM-RPE thickness, an established biomarker of mitochondrial respiration (see above), is unclear.

In this study, we tested the hypothesis that the HB magnitude is modulated by mitochondria respiration. To examine this question, HB magnitude and ELM-RPE thickness were compared following two drug treatments: (i) acetazolamide (ACZ), a carbonic anhydrase inhibitor that acidifies the subretinal space and triggers ELM-RPE thinning in light-adapted mice^13,35–38^, and (ii) 2,4-dinitrophenol (DNP), a well-studied mitochondrial uncoupler that stimulates respiration at non-toxic doses^39–41^ and significantly reduces ELM-RPE thickness in light-adapted mice^30^. In addition, effects of DNP on histology cell structure in the HB region were examined using F-actin distribution as an index, and on phototransduction using ERG recordings. Further, as C57BL/6J mice have relatively inefficient mitochondrial respiration compared to 129S6/ev mice^15,31^, mice with different strains (genetic background) were used for testing mitochondrial function on light–dark changes in HB magnitude and ELM-RPE thickness. Finally, we looked for proof-of-concept evidence regarding whether HB magnitude also varied with light and dark conditions in humans based on previously published data captured using a commercial instrument^33^. Our results indicate that HB magnitude represents a novel OCT biomarker of mitochondria respiration in human and mouse retina that is upstream of pH-triggered changes in ELM-RPE thickness.

## Results

### Light–dark changes of HB in outer retina

**Figure 1:**
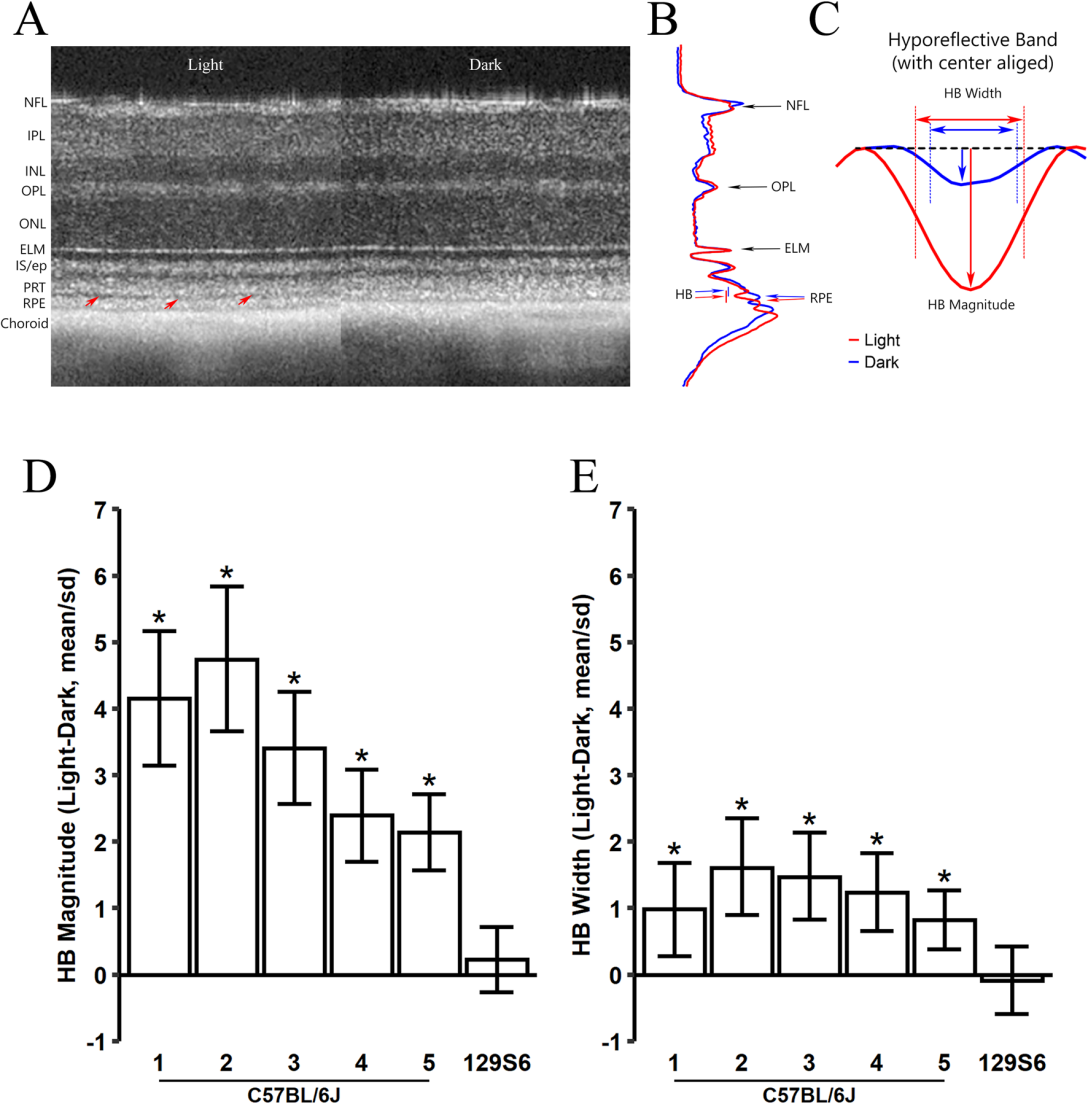
Effect of light and dark adaptation on the HB in outer retina. **(A)** Example of OCT images captured in light and dark from the same mouse eye. Red arrows point the HB between photoreceptor tip (PRT) and retinal pigment epithelium (RPE) layers. *NFL* nerve fiber layer, *IPL* inner plexiform layer, *INL* inner nuclear layer, *OPL* outer plexiform layer, *ONL* outer nuclear layer, *ELM* external limiting membrane, *IS/ep* inner segment/ellipsoid layer. **(B)** Representative intensity profiles averaged from all four radial scans of the eye in **(A)** for images captured in light (red) and dark (blue). HB is marked by a bar on left side. **(C)** Magnified view of HB. The dashed line represents the baseline constructed by connecting the maximal intensity for photoreceptor tip layer and RPE layer (Supplemental Figure S2) and rotated to horizontal. The intensity profiles are shifted laterally with center aligned for light and dark conditions. HB magnitude is calculated as the peak distance from the baseline line; HB width is measured as length between two half maximal points. Normalized light–dark responses of HB magnitude **(D)** and width **(E)** were made from 5 C57BL/6J mouse groups (Table 1) and one 129S6/ev group under light and dark conditions. Values are normalized by standard deviation of each mouse group. An asterisk indicates the credibility interval excludes 0 (equivalent to statistically significant).

The regions-of-interest are shown on a paired set of full-frame OCT B-scan images obtained after light and dark adaptation (supplemental Figure S1, and shown side-by-side in Fig. 1A). Light-adapted retinas show an HB (red arrows) between rod photoreceptor outer segment tips (PRT) and apical RPE^3,14,15^. Transretinal intensity profiles are shown in Fig. 1B under the two conditions, with HB region marked by a bar on the left side (red for light-adapted curve and blue for dark-adapted curve). For visual comparison of the light–dark differences, centrally aligned HB regions are shown in Fig. 1C. To quantitate HB magnitude, a baseline was constructed as a straight line connecting maximum intensity of PRT layer and the RPE layer (shown as a dashed line in supplemental Figure S2). HB magnitude is measured as the absolute value of the peak amplitude from baseline, and width is measured as length between two half maximum points on each arm of the profile.

Dark adaptation significantly reduced both HB magnitude (raw data presented in supplemental Figure S3A) and width (raw data presented in supplemental Figure S3B, and examples of light/dark pair of OCT images shown in supplemental Figure S4) for all C57BL/6 J mice groups (Table 1), but HB magnitude and width did not decrease in the dark for the 129S6/ev group. Light–dark changes in HB magnitude and width are shown in Fig. 1D,E, respectively. To facilitate comparison, responses were normalized to their standard deviations and plotted on the same scale. For C57BL/6J mice groups, the light–dark HB magnitude showed a large change in both absolute scale (averaging 5.6 to 12.7 units on standard 256 unit (8-bit) grayscale images, Fig S3A) and relative to the within-group standard deviation (> 2 mean/sd, Fig. 1D). In contrast, the light–dark HB width changes were small on an absolute scale (< 2 µm, or about 1 pixel on the OCT image, of averaged differences for all mouse groups, Fig S3B) and relative to the within-group standard deviation (< 2 mean/sd, Fig. 1E). Thus, the relatively larger signal-to-noise ratio of light–dark HB magnitude was considered the outcome index for the rest of this study.

**Table 1:** Mouse groups used for OCT studies.

Group	B6-1	B6-2	B6-3	B6-4	B6-5	S6	Nob2+	Nob2−
N	4	4	5	6	10	8	6	5
Strain	C57B6					129S6/ev	AXB6/B6	
Gene	Nob7+	Nob7−	WT	WT	WT	WT	Nob2+	Nob2−
Drug			DNP	DNP	ACZ			
Dose			10 mg/kg	5 mg/kg	250 mg/kg			

### Light–dark HB magnitude and ELM-RPE thickness changes show distinct responses to pH

**Figure 2:**
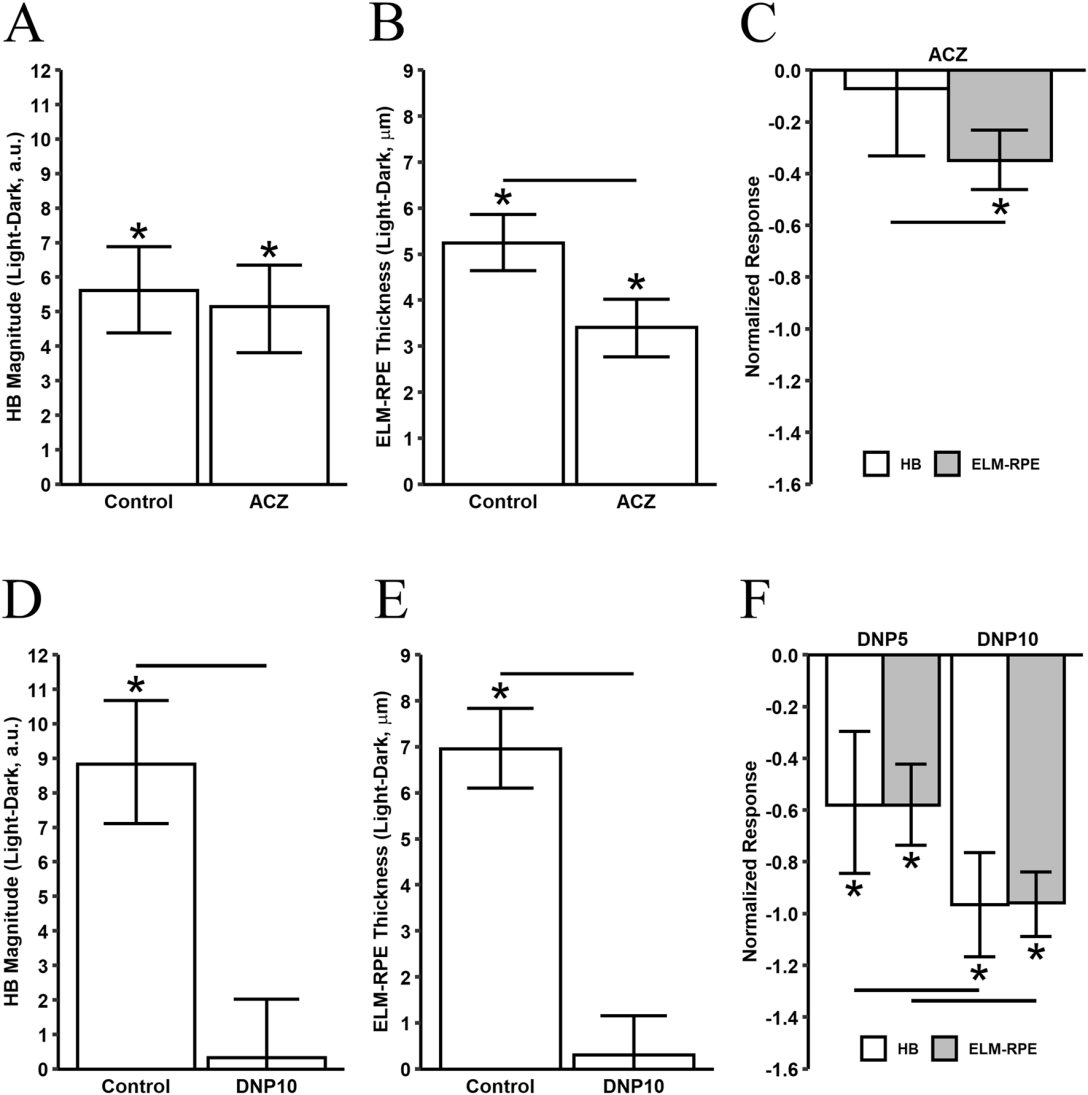
Effect of ACZ and DNP on outer retina responses. Light–dark difference of HB magnitude **(A,D)** and ELM-RPE thickness **(B,E)** for control (baseline) and after ACZ and DNP (10 mg/kg) treatments, respectively. **(C,F)** Normalized drug response (${\tilde{Y }}_{drug}- {\tilde{Y }}_{control})/{\tilde{Y }}_{control}$ in light–dark differences ($\tilde{Y }$ represents the mean light–dark difference) in HB magnitude and ELM-RPE thickness for control and mice treated with ACZ and DNP (at two dosage, 5 and 10 mg/kg). All error bars represent 95% credibility intervals, an asterisk indicates the credibility interval excludes 0 (equivalent to statistically significant), and a line between two bars indicates that the credibility interval of the difference between the two estimates are statistically significant. (ACZ, n = 10 mice; DNP at 10 mg/kg, n = 5 mice; DNP at 5 mg/kg, n = 6 mice).

Acetazolamide (ACZ) is a broad-spectrum carbonic anhydrase inhibitor that acidifies the SRS^35–38^. Here, we compare the effects of ACZ on light–dark changes in HB magnitude and ELM-RPE thickness. ACZ produced no significant effect on light–dark changes in HB magnitude (Fig. 2A with raw data presented in supplemental Figure S5A). On the other hand, ACZ produced a thinner ELM-RPE for light-adapted retina (Fig. 2B with raw data presented in supplemental Figure S5B, and averaged data in supplemental Figure S5C) consistent with previous observations that carbonic anhydrase inhibitors prevent the light-evoked water increase in subretinal space^13,42^. Further, direct comparison of light–dark changes in the ELM-RPE thickness with HB magnitude (both normalized to their respective pre-treatment values; Fig. 2C) was statistically significantly. These results raise the possibility that light–dark changes in HB magnitude are pH-independent and upstream of pH-triggered thinning of the ELM-RPE region.

### Increasing mitochondrial activity induced parallel reductions for light–dark changes in HB magnitude and ELM-RPE thickness

Next, we directly tested for a role of mitochondrial respiration, a major contributor for light-dependent water productions in the retina, in modulating HB magnitude. Mitochondrial activity was specifically increased using non-toxic doses of the protonophore 2,4 dinitrophenol (DNP) on the light–dark changes in HB magnitude and ELM-RPE thickness. DNP significantly reduced light–dark HB magnitude (Fig. 2D with raw data presented in supplemental Figure S6A). As expected, light–dark changes in ELM-RPE thickness was also reduced with DNP (Figs. 2E with raw data presented in supplemental Figure S6B)^30^. Mice treated with 5 mg/kg DNP had ~ 60% reduction of light–dark changes in both the HB magnitude (normalized DNP response = − 0.579, 95% credibility interval: − 0.828, − 0.295) and ELM-RPE thickness (normalized DNP response = − 0.580, 95% credibility interval: − 0.736, − 0.426). Mice treated with 10 mg/kg DNP showed more than 90% reduction of both OCT responses from their respective controls (HB magnitude: − 0.962, 95% credibility interval: − 1.166, − 0.765; ELM-RPE thickness: − 0.957, 95% credibility interval: − 1.082, − 0.834). These results indicate that mitochondrial respiration underlies both the HB magnitude and ELM-RPE thickness (Fig. 2F).

### DNP had no effect on ERG responses

**Figure 3:**
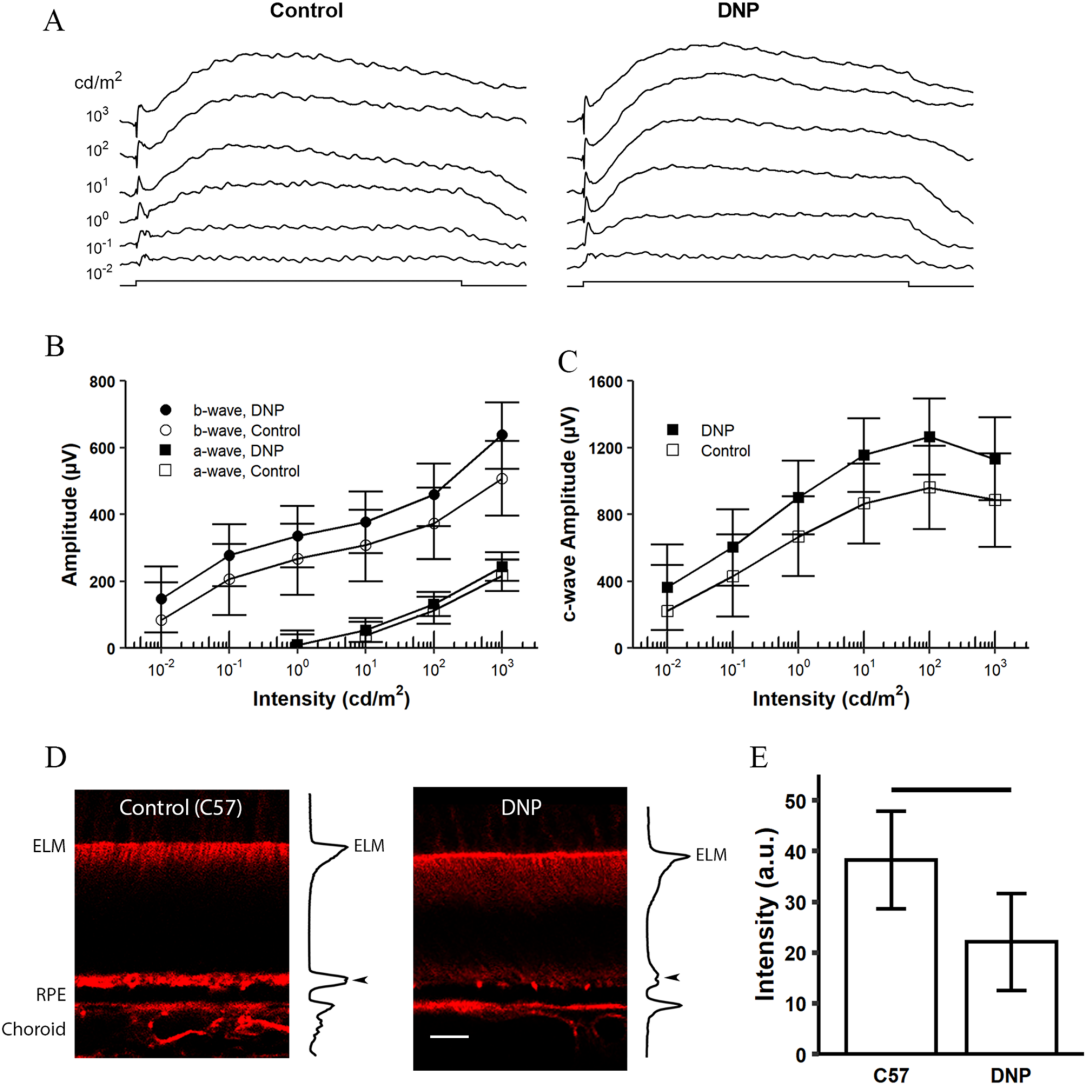
Effect of DNP on ERG responses and on F-actin distribution in outer retina. **(A)** Representative ERG waveform recorded from a saline-injected C57BL/6J mouse (control, left panel) and a DNP (10 mg/kg) injected C57BL/6J mouse (right panel). The light stimulus (10 s) is shown in the bottom trace. Intensities of stimulus light are shown on left side of the panel. Amplitudes of the ERG a- and b-wave **(B)** and c-wave **(C)** obtained from control (n = 5 mice) and DNP treated (n = 5 mice) mice. **(D)** Example of F-actin distribution based on phalloidin staining pattern in outer retina of light-adapted (Control) and DNP-treated (DNP) C57B6/6J mice. Scale bar: 10 µm. Fluorescent intensity profiles are plotted on right side of each image. Arrow heads point to the peak intensity at HB region. **(E)** Mean peak phalloidin fluorescent intensity measured at HB region (Control, n = 6; DNP, n = 6). Error bars represent 95% credibility intervals, and a line between two bars indicates that the credibility interval of the difference between the two estimates are statistically significant.

We used ERG recordings to probe effects of DNP on phototransduction and movement of ions in subretinal space, and to test the possibility that the DNP-evoked decrease in HB magnitude was due to altered retinal function. Dark-adapted ERG a-wave reflects phototransduction responses in rod photoreceptors and c-wave is mediated by the RPE and Müller cells in response to potassium ion concentrations in the SRS. Figure 3A shows examples of ERG waveform elicited from saline (Control) and DNP (10 mg/kg) treated mice to 10 s light flashes at various intensity shown on the left side panel. The average intensity-response relationship for a- and b-wave obtained from DNP-treated and control mice are shown in Fig. 3B. The a-wave amplitudes from DNP-treated mice were not statistically different to those of control mice, indicating rod photoreceptor activity in the retina was not significantly affected by DNP. DNP-treated mice exhibited slightly but not significantly larger b-wave amplitudes than those of controls (Fig. 3B). The estimated intensity-response relation of c-wave responses showed ~ 10% increased amplitude with DNP treatment across all intensities, but the credibility intervals for DNP and control overlap at all light stimulus intensities tested (Fig. 3C). These results indicate that the light–dark changes in HB magnitude and ELM-RPE thickness are not due to DNP toxicity and are functional indices distinct from ERG parameters.

### DNP reduced F-actin band in HB region

We used F-actin/phalloidin staining as an index to study cellular structural changes in the HB region. Figure 3D shows examples of F-actin distribution pattern for light-adapted C57BL/6 J (served as controls) and DNP-treated mice (which showed significant lower HB magnitude and ELM-RPE thickness, Fig. 2D–F). The intensity profiles of phalloidin staining are shown on the right side of each photograph with arrow heads indicating the HB region. Phalloidin staining at the HB region can be visually appreciated in control light-adapted mice, whereas staining was diminished in DNP-treated mice which resembled mice after dark-adaptation^3^. Quantitative analyses support these impressions. Average F-actin fluorescent intensities at the HB region are shown in Fig. 3E. F-actin intensity of DNP-treated mice was significantly lower than that of controls (95% credibility interval for the difference: − 29.7, − 2.8). Mice given saline injections exhibited the same pattern as un-injected controls (Supplemental Figure S7). Together, these results support and extend our previous observation that the light–dark changes in HB magnitude are correlated with light-dependent redistribution of F-actin^3^.

### Correlations of light–dark changes in HB magnitude with ELM-RPE thickness

**Figure 4:**
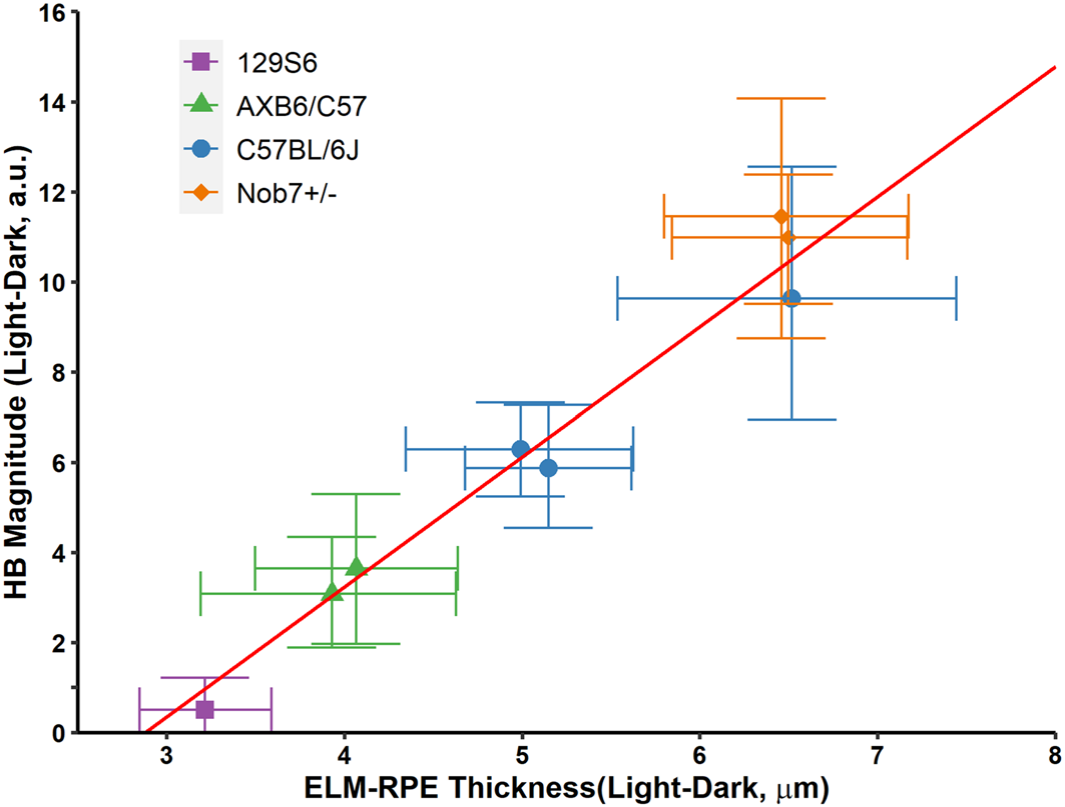
Correlation of light–dark HB magnitude with ELM-RPE thickness changes. Responses were obtained from 8 mouse groups (Table 1). The red line represents the significant association estimated by the Bayesian hierarchical model (the between-group correlation converted to a regression coefficient). All error bars represent 95% credibility intervals.

We next took advantage of the fact that mitochondrial respiration is strain-specific to further test the notion that photoreceptor mitochondria metabolic activity modulates both OCT indices by investigating the relationship between HB magnitude and ELM-RPE thickness. Five strains were examined: C57BL/6J, Nob7- and Nob7 + on C57BL/6J backgrounds, Nob2 KO on a AXB6/B6 background (and wildtype littermates), and 129S6^15^ (see Table 1, and examples of light/dark OCT image pairs in supplemental Figure S4). We examine the between-group correlation between the light–dark responses in HB magnitude ad ELM-RPE thickness. Nob2 mice with mixed genetic background of AXB6/B6 have intermediate levels of ELM-RPE changes compared to C57BL/6J and 129S6/ev groups. Results are shown in Fig. 4 with a significant correlation (r = 0.975, 95% credibility interval: 0.756, 1.000). These results suggest a link between light–dark HP magnitude and ELM-RPE changes among strains, which further support the notion that light–dark differences in mitochondria respiration modulates the HB magnitude.

### Light–dark changes of HB in human retina

**Figure 5:**
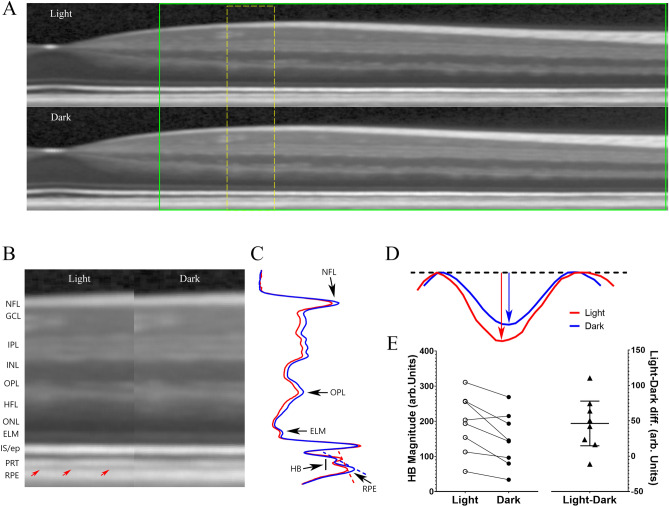
Effect of light and dark adaptation of HB magnitude on human subjects. **(A)** Representative OCT images from a human eye captured under light and dark conditions. Images were flatted along RPE band. Regions marked by green box outline images used for calculating HB magnitude. **(B)** Magnified OCT images from region outlined by yellow dash line in **(A)** showed side-by-side. The location of the HB is marked by red arrows for the light-adapted retina. **(C)** Intensity profiles calculated from images shown in **(B)** with HB region marked by a bar on left. The dashed lines represent the baseline constructed by connecting the maximal intensity for photoreceptor tip layer and RPE layer. **(D)** Magnified view of HB with the baselines rotated and aligned. HB magnitude is measured as the peak distance from the baseline line. **(E)** HB magnitude under light and dark conditions for 8 subjects (left panel) and Light–dark difference of HB magnitude (right panel). Error bar represents 95% credibility interval. Please note that different hardware and grayscale outputs were used for OCT images collected for human and mouse studies.

A HB between photoreceptor outer segment tips and apical RPE has been observed in human OCT images, especially in the parafovea area^7,32,33^. However, functional responses of the HB to light and dark in human subjects have not been investigated. To begin to address this question, we analyzed a set of OCT images from a previously published study^33^ collected under shorter light- and dark-adapted conditions than from our mouse studies and that reported light-induced reflectivity changes in retinal layer. An example of paired OCT B-scan images from the same human subject obtained after light- and dark-adaptation are shown in Fig. 5A. For visual comparison, light–dark changes in a magnified portion of the OCT image in Fig. 5A marked by yellow dash lines are shown side-by-side in Fig. 5B. We focused our analysis on the HB that can be visually appreciated from both light-adapted (red arrows) and dark-adapted images (but that appears less prominent after dark-adaptation). Figure 5C illustrates intensity profiles calculated from images shown in Fig. 5B. The HB region is marked by a bar on the left side of the intensity profile. A baseline was constructed as a straight line connecting maximum intensity of photoreceptor tip layer and the RPE layer (shown as a dashed line in Fig. 5C). The HB is defined as the difference between OCT intensity curve and the baseline, and a magnified view shown in Fig. 5D. HB magnitude is measured as the peak difference between OCT intensity and the baseline. Figure 5E summarizes results obtained from 8 young adults. The paired light–dark difference of HB magnitude was significant (p = 0.01). These results provide proof-of-concept evidence for light-evoked changes in HB for human subjects, in addition to other OCT responses reported previously^33^.

## Discussion

**Figure 6:**
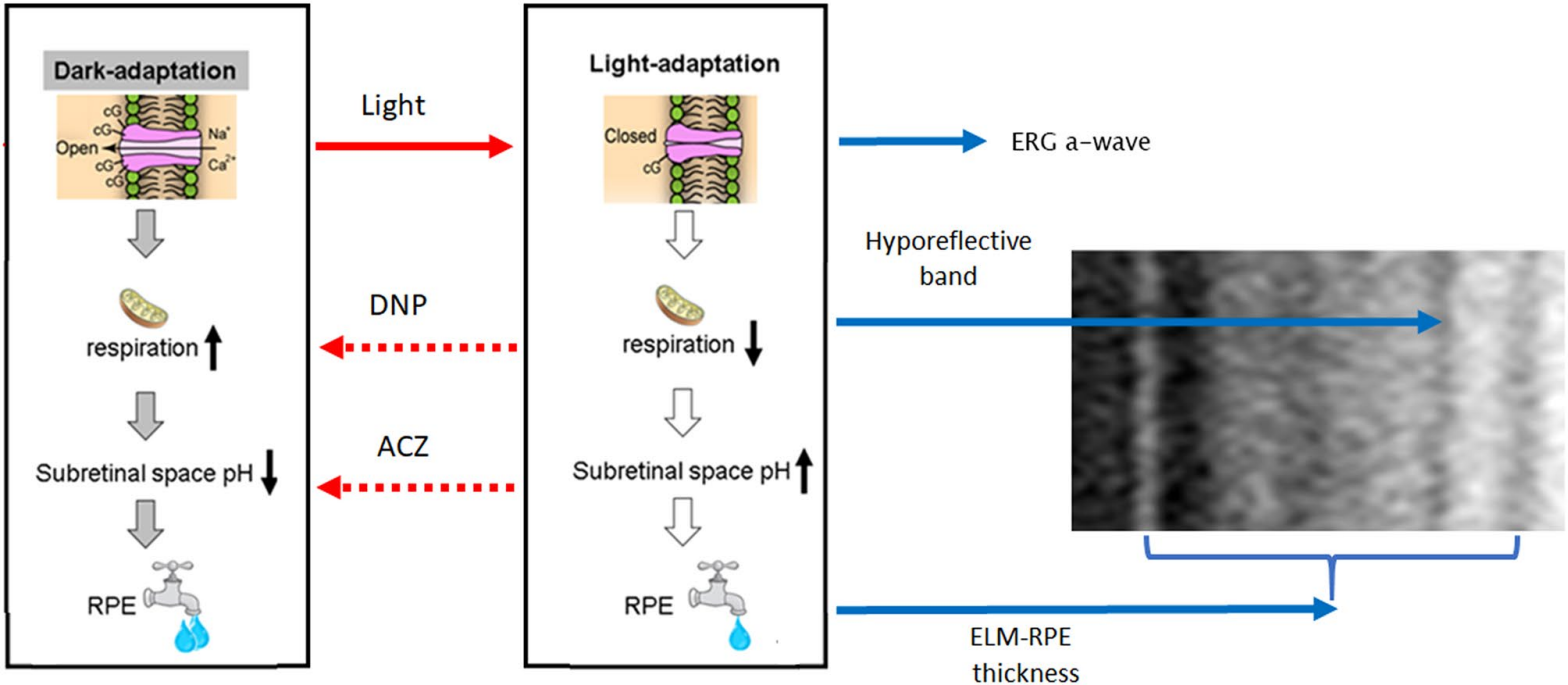
Proposed signaling pathway for two light-induced OCT responses. Adding either DNP or ACZ converted the light phenotype (middle panel) to a dark phenotype (left panel). Our data suggests that DNP activates the upstream mitochondrial signal inducing changes in HB magnitude and ELM-RPE thickness (right panel); ACZ presumed impact on pH was limited to ELM-RPE thickness changes. Based on these observations, we propose that the light–dark HB magnitude change reflects mitochondrial function, whereas the ELM-RPE represents both mitochondrial function and its downstream effect on subretinal space pH.

In this study, we report first-time support for underlying mechanisms for functional changes in the HB magnitude in the mouse with initial supporting data in humans. In the dark, metabolic activity of rod photoreceptors is greater than in the light, causing a relative increase in the production of lactate, CO_2_ and waste water (compared to that in light)^16,21–23,43^. Together, these factors acidify the subretinal space, triggering an increase in co-transporter-based water removal by the RPE, and significant shrinkage of the ELM-RPE region^16,21–23,43^. Indeed, HB magnitudes were shown to be modulated by DNP-induced increases in photoreceptor mitochondria activity (Fig. 2D–F). On the other hand, HB signal was not affected by acidification of the SRS, as ACZ had no effect on HB magnitude but significantly reduced ELM-RPE thickness (Fig. 2A–C). These data suggest a working model in which HB magnitude is regulated by mitochondria upstream of pH-triggered changes in ELM-RPE thickness. Figure 6 illustrates a working model, formulated from all of the available evidence in this study and the literature, to underscore where the two novel OCT biomarkers fit in the energy ecosystem (i.e., mitochondria function and its downstream consequences at the rod / RPE interface) of the outer retina.

This model may also be useful in explaining the inverse correlation between the efficiency of mitochondria and the magnitude of the change in HB in, for example, C57BL/6J mice. The less efficient mitochondria of C57BL/6J rod mitochondria produce more waste water and larger HB than that of the more efficient mitochondria of 129S6/ev mice^15^. Also, in the dark or following exposure to DNP, RPE fluid transport is more active relative to that in the light reducing SRS water content and HB^14^. These observations suggest the hypothesis that variations of SRS water content contribute to the HB changes in light and dark. In line with this notion, mice of mixed C57BL/6J and AXB6 background had an intermediate light–dark HB (Fig. 4).

Mutants in the Nob2 (No B-wave type 2) gene that affects synaptic transmission by photoreceptors^44^ have minimal impact on light-evoked OCT structural changes, as this mutant mouse strain showed similar responses as wildtype littermates. This conclusion was further supported in Nob7 mutants (Figs. 1 and 4, Table 1) which carries a mutation in the mGluR6 gene that blocks ON pathway^45^. These results suggest that the inner retina circuitry was not a major contributor to either HB magnitude or the ELM-RPE thickness changes between the light- and dark-adapted conditions.

We considered the possibility that the correlation between light–dark ELM-RPE and HB changes was due simply as a result of the larger light–dark ELM-RPE thickness changes. However, this does not explain the disconnect between the two indices following ACZ (Fig. 2A–C) nor in the 129S6/ev mice (Figs. 1 and 4). Intriguingly, human studies that used a different light–dark protocol than the mouse studies show significant reduction of HB from light to dark (Fig. 5) but no change in ELM-RPE thickness^33^. At present, the structural basis for the HB in human OCT is unclear^46–49^. In this study, we found reduction in the HB induced either pharmacologically (DNP) or by dark-adaptation^3^ is associated with a reduction of F-actin in this region (Fig. 3D,E), in agreement with reports of other macromolecule changes in region^50–52^. Our results are consistent with modulation of F-actin signal initiated from mitochondrial activity, as water has a substantial impact on cytoskeleton dynamics^53^. However, the precise pathway leading to actin polymerization and its link to the OCT HB signal remains to be elucidated. Nonetheless, together, these results support the hypothesis that HB and ELM-RPE thickness are two distinct but correlated functional biomarkers.

Another possible confounding factor is that HB magnitude reflects localized or whole retinal changes in reflectivity that can occur between light and dark^8,24,33^. To test for this, we first show that light–dark changes in HB magnitude (Fig. 5) was unrelated to the changes on PRT layer (OStip) reflectivity (Supplemental Figure S8)^33^. Similarly, there are also no consistent changes in OCT intensities for the RPE and PRT layers for OCT images captured from light- and dark-adapted mouse retinas (Supplemental Figure S9A and B). Also, there is no consistent change in OCT intensity for the IS/ep layer (Supplemental Figure S9C). These data support the notion that HB is a unique feature within the OCT that is not simply mirroring more widespread changes in reflectivity. Recently, it has been suggested that light-induced OCT intensity increase for the IS/ep layer may be linked to mitochondria activity of the photoreceptor^24^. However, those changes were at a much faster time scale, and more work is needed to determine if they are mediated by different mechanisms than the OCT responses described in this study.

Intriguingly, we find that DNP at 10 mg/kg almost completely eliminated the light–dark changes measured by OCT (Fig. 2D–F) but surprisingly had no discernable effect on the ERG amplitudes (Fig. 3A–C). By way of explanation, we first note that a relatively sizable mitochondria reserve is available for the light-adapted retina^15^. Low dose of DNP as used in this study would increase metabolic rate and produce more CO_2_ and waste water. Increased metabolic activity reduces pH level in the SRS and stimulates fluid removal by RPE, a situation similar to dark-adapted retina. Nonetheless, normal ERG responses after DNP treatment (Fig. 3A–C) suggest no significant alteration of ATP levels in the photoreceptors. Dark-adapted ERG a-wave is a non-invasive measure of rod phototransduction; c-wave reflects a combination of RPE and the Muller cell activity in response to potassium ions in the subretinal space^54,55^. However, in dark-adapted C57BL/6J mice, mitochondrial reserves are low and ELM-RPE thickness is unresponsive to DNP^14,30^. Nonetheless, light–dark changes for both HB and ELM-RPE thickness are diminished following DNP (Fig. 2D–F).

In summary, we provide evidence that the light–dark changes in HB magnitude, ELM-RPE thickness, and ERG recordings probe different functional aspects of the outer retina (e.g., Fig. 6). The OCT biomarkers, unlike ERG, do not require assumptions of unchanging retinal resistance to interpret functional changes. We note that the largest light–dark change in HB magnitude was seen in C57BL/6J mice which has a relatively less efficient mitochondria, raising the possibility that a large light–dark HB magnitude response is an index of poorly regulated mitochondria; more work is needed to test this notion^15^. If this hypothesis is proved to be true and could also be extended to human, HB might indicate regions of high susceptibility to damage, an intriguing possibility that warrants further investigation. HB can be detected on human OCT images captured with commercial instruments commonly used in eye clinic^7,32,33^. These considerations support future animal and human studies measuring HB magnitude, together with the ELM-RPE thickness, as a functional biomarker “tool kit” for diagnosing and treating various photoreceptor mitochondria-based retinopathies.

## Methods

### Mouse strains

All of the procedures involving animals were conducted under an approved NIH animal care protocol by National Eye Institute Animal Care and Use Committee and ARRIVE guidelines. All methods were performed in accordance with the relevant guidelines and regulations. All animals of both sexes were kept in regular animal housing under a 50 lx 14:10 h dark–light cycle. Mouse groups used for OCT studies are listed in Table 1. C57BL/6 J mice were obtained from Jackson Labs (Bar Harbor, ME), 129S6/ev mice were obtained from Taconic Labs (Rensselaer, NY), Nob2 mice in an AXB6/C57 background (Nob2-) were purchased from the Jackson Laboratory and crossed back to C57BL/6J for five generations (ABX6/B6). In addition, Nob7 mutant mice of C57BL/6J strain^45^ and their littermate were also used.

### OCT imaging and analysis

OCT images were captured using an Envisu UHR2200 (Bioptigen, Durham, NC) with theoretical axial resolution in tissue of 1.6 µm. Mice were anesthetized with ketamine (100 mg/kg) and xylazine (6 mg/kg), eye was positioned with the optic nerve head (ONH) in the center of the OCT scan. Each mouse was scanned twice: first between 5 and 7 h after exposure to room light mice under standard illumination conditions (~ 500 lx), second, mice were then dark-adapted overnight and rescanned the next day in darkness but otherwise under the same conditions as above. In all cases, four radial scans (step at 45-degree intervals each with 40 times averaging, 1. 4 mm at 1000 A-scan × 4 B-scan × 40) were captured. As a convention for commercial OCT system, images were generated on logarithmic scale. The averages of the four radial scan OCT images were used to measure retinal layer thickness. An example full-frame OCT images captured in light and dark from the same mouse eye is shown in supplemental Figure S1. HB magnitude and width using a custom made Matlab program. Briefly, OCT images of 350 µm to 630 µm away from optic nerve head (delineated by two green dash lines in supplemental Figure S1) were analyzed. Each image was binned every 10 pixels, OCT transretinal intensity profiles were aligned at the ELM peaks and averaged among 4 radial scans. Outer retina length was measured from ELM to basal side of RPE layer, two clearly distinguishable OCT layers. To measure HB of the outer retina, a straight line was constructed between maxima of the photoreceptor tip and RPE layers and served as the baseline. HB is defined as the difference between this baseline and OCT intensity profile. Peak amplitude from baseline served as HB magnitude, and length between two half maximum points served as HB width. See Fig. 1 and supplemental Figure S2 for details.

### Systemic drugs studies

Effects of systemic administration of drugs on OCT responses were investigated with C57BL/6J mice. All drugs were given at a volume of 10 µl/g of body weight by i.p. injections. 2,4-Dinitrophenol (DNP) and acetazolamide (ACZ) were purchased from Sigma-Aldrich (St. Louis, MO). DNP was dissolved in PBS (pH 7. 2) at 0.5 or 1 mg/ml and given to mice at 5 or 10 mg/kg. DNP is protonophore that stimulates mitochondrial respiration at these non-toxic doses^30,41^, and OCT images were captured 1 h after injection. ACZ was dissolved in pH 9 phosphate buffer saline (PBS) to a final concentration of 25 mg/ml, and given to mice by i. p. injection at a dose of 250 mg/kg, and imaged 3 h after injection^56^. ACZ acidifies the subretinal space and suppresses the light–dark ELM-RPE difference as measured by diffusion MRI^22,57,58^. Given the variability between different mouse groups in OCT responses, as shown in the Results section, we first measured baseline dark–light OCT images with mice given PBS injections (pH 7. 2 PBS for DNP groups and pH 9 PBS for ACZ group). After 3 days rest, dark–light OCT images were captured again after receiving drug treatment. In a preliminary experiment, a separate group of mice were imaged at baseline without any injection, and 3 days later with PBS injection. No significant differences were detected with PBS injection.

### Immunohistochemistry

To measure F-actin distribution, mice were intracardially perfused with 4% paraformaldehyde in PBS after deep anesthesia. Eyes were enucleated and then immersed in additional fixative for one hour. Posterior eye cups were embedded in 4% agarose (Sigma-Aldrich Corp., St. Louis, MO, USA) and sectioned at 100 µm with a vibratome. Sections were washed with PBS containing 0.1% Triton X-100 and blocked overnight with PBS containing 1% BSA. Sections were incubated for 24 h with Alexa Fluor 546 phalloidin (Invitrogen) containing 1% BSA. After washing 3 times with PBS, the sections were stained with DAPI, mounted, and imaged on a Zeiss confocal laser scanning module LSM 510 Microscope System.

### Electroretinogram (ERG) recording

The recording procedure of the ERG followed published protocols^59^. Briefly, following over-night dark adaptation, mice were anesthetized with ketamine (100 mg/kg) and xylazine (6 mg/kg), and eyes dilated with a drop of tropicamide and phenylephrine. Body temperature of the mouse was maintained at 37 °C by a heating pad. Electroretinograms were recorded with an Espion E2 Visual Electrophysiology System (Diagnosys, Lowell, MA, USA) from both eyes using gold wire loop electrodes with band pass filtering from DC to 300 Hz. A 10-s step of light with intensity range from 0. 01 to 1000 cd/m^2^ was delivered to mouse eye by the attached ColorDome. Inter-stimulus intervals were 1 to 5 min depending on stimulus intensity. A-wave amplitudes were measured from baseline to negative trough, and b-wave from trough to positive peak. Slow developing c-wave amplitudes were measured from baseline to the peak usually occurring around 3–5 s after light onset.

### Human OCT image analysis

A previously published OCT dataset (publicly available in the data repository: https://doi.org/10.5061/dryad.msbcc2ftc) was used to measure HB in human subjects. The original study was approved by the Oregon Health and Science University (OHSU) IRB and informed consent was obtained from all subjects prior to the study^33^. Briefly, images were captured using a commercial instrument (Spectralis, Heidelberg Engineering, Franklin, MA) after 20 s light exposure (Light) and after 2 min in dark (Dark). Images were averaged for 15 such light/dark cycles, flattened according to RPE band, and resampled to spatially normalize retinal bands (see^33^ for details). HB magnitude was calculated using the method described above for mouse OCT images.

### Statistical analysis

We used Bayesian analyses for all outcomes. As such, we present results as estimates (means or differences in means) ± 95% credibility interval, where a 95% credibility interval has a 95% probability of containing the true value of the quantity being estimated given the data. (In contrast, a 95% confidence interval is an interval that has 95% confidence that the true value would be in the interval, with 95% of all constructed confidence intervals, over a large number of experiments, containing the true value.) We used Markov Chain Monte Carlo (MCMC) for fitting all of the models. This technique simulates the distribution of the parameter estimates, with several simulations (chains) being run independently. All analyses simulated four chains with 30,000 iterations, and thinning by 5, for each model. We used RStan^60,61^ to fit models for the following analyses: examining the differences in HB magnitude and HB width among groups, estimating the correlation between HB magnitude and ELM-RPE thickness, examining differences in F-actin and ERG. The analysis of drug effects on HB magnitude and ELM-RPE thickness was fit using PROC BGLIMM in SAS 9.4 (SAS software, Cary, NC, USA). We constructed 95% credibility intervals based on the observed percentiles of the simulated posterior distributions for all relevant parameters. Credibility intervals for all estimates and comparisons were calculated from the parameter samples.

Light–dark responses in HB magnitude and HB width were modeled using a model having an intercept representing the overall mean with a diffuse normal prior, the group means having a hierarchical prior based on the overall mean and between-group variance, and both the within- and between-group variances having diffuse Cauchy distributions.

Light–dark response in ELM-RPE thickness and in HB magnitude were first modeled for the mouse groups not receiving any drug treatment. For this analysis, we calculated the light–dark differences for each mouse and used this light–dark difference as the dependent variable in the model. We jointly modeled the light–dark difference in ELM-RPE thickness and the light–dark difference in HB magnitude. We assumed that the light–dark difference for HB magnitude and the light–dark difference for ELM-RPE thickness for the $i$th mouse within-group $j$ were multivariate normally distributed,

$${{\left[\begin{array}{cc}{Y}_{ELM-RPE}& {Y}_{HB}\end{array}\right]}_{i}}^{T}\sim MVN\left({\left[\begin{array}{cc}{\mu }_{ELM-RP{E}_{j}}& {\mu }_{H{B}_{j}}\end{array}\right]}^{T}, {{\varvec{\Sigma}}}_{{\varvec{j}}}\right)$$

with the mean and covariance matrix being determined by the group. We used a hierarchical prior for the mean vector $${{\varvec{\mu}}}_{{\varvec{j}}}=\boldsymbol{ }{\left[\begin{array}{cc}{\mu }_{ELM-RP{E}_{j}}& {\mu }_{H{B}_{j}}\end{array}\right]}^{T}$$, ${{\varvec{\mu}}}_{{\varvec{j}}}\sim MVN\left({\varvec{\mu}}, {{\varvec{\Sigma}}}_{{\varvec{\mu}}}\right)$, where the overall mean vector $${\varvec{\mu}}={\left[\begin{array}{cc}{\mu }_{ELM-RPE}& {\mu }_{HB}\end{array}\right]}^{T}$$ has independent normal priors, ${{\varvec{\Sigma}}}_{{\varvec{\mu}}}$ is the between-group covariance matrix, the variances of ${{\varvec{\Sigma}}}_{{\varvec{\mu}}}$ have independent Cauchy priors, and the between-group correlation among means has a beta prior, ${{r_{\mu } + 1} \mathord{\left/ {\vphantom {{r_{\mu } + 1} 2}} \right. \kern-\nulldelimiterspace} 2}\sim beta\left( {0.5,0.5} \right)$. We also used hierarchical priors for the within-group covariance matrix ${{\varvec{\Sigma}}}_{{\varvec{j}}}$ in which each of the variances has a lognormal distribution $\left[{\sigma }_{k}^{2}\sim lognormal\left({\mu }_{{\sigma }_{k}}, {\sigma }_{{\sigma }_{k}^{2}}^{2}\right)\right]$ and the within-group correlation for each group, ${r}_{j}$ has a beta distribution dependent on the overall mean correlation, $r$, ${{r_{j} + 1} \mathord{\left/ {\vphantom {{r_{j} + 1} 2}} \right. \kern-\nulldelimiterspace} 2}\sim beta\left( {{{r + 1} \mathord{\left/ {\vphantom {{r + 1} 2}} \right. \kern-\nulldelimiterspace} 2},\sigma _{r}^{2} } \right)$, using the mean and variance parameterization of the beta distribution. We used Cauchy hyperpriors for the mean variances and the variances of the mean variances. We used a beta(0.5, 0.5) hyperprior for ${{r + 1} \mathord{\left/ {\vphantom {{r + 1} 2}} \right. \kern-\nulldelimiterspace} 2}$ and a $uniform\left( {0,\left( {{{r + 1} \mathord{\left/ {\vphantom {{r + 1} 2}} \right. \kern-\nulldelimiterspace} 2}} \right)\left( {1 - {{r + 1} \mathord{\left/ {\vphantom {{r + 1} 2}} \right. \kern-\nulldelimiterspace} 2}} \right)} \right)$ hyperprior for ${\sigma }_{r}^{2}$. We used Cholesky decompositions of the covariance matrices.

A bivariate linear mixed model^62,63^ was used to jointly model light–dark changes in HB magnitude and ELM-RPE thickness using Proc BGLIMM in SAS. The calculated light–dark change for both HB magnitude and ELM-RPE thickness were used as the dependent variables in this joint model that also included the fixed effects of drug treatment (no vs yes), drug-treatment group (ACZ, 5 mg/kg DNP, and 10 mg/kg DNP), and the drug treatment by drug-treatment group interaction:

$${HB}_{ij}=\left({\beta }_{H0}+{u}_{Hi}\right)+{\beta }_{H1}*{treat}_{ij}+{\beta }_{H2}*{DNP5}_{i}+{\beta }_{H3}*{DNP10}_{i}+{\beta }_{H4}*trea{t}_{ij}*{DNP5}_{i}+{\beta }_{H5}*trea{t}_{ij}*{DNP10}_{i}+{\epsilon }_{Hij},$$

$${ELM-RPE}_{ij}=\left({\beta }_{ER0}+{u}_{ERi}\right)+{\beta }_{ER1}*{treat}_{ij}+{\beta }_{ER2}*{DNP5}_{i}+{\beta }_{ER3}*{DNP10}_{i}+{\beta }_{ER4}*trea{t}_{ij}*{DNP5}_{i}+{\beta }_{ER5}*trea{t}_{ij}*{DNP10}_{i}+{\epsilon }_{ERij},$$

with $H{B}_{ij}$ being the light–dark difference in HB magnitude for the $j$th observation of mouse $i$, ${ELM-RPE}_{ij}$ being the light–dark difference in ELM-RPE thickness, $${\left[\begin{array}{cc}{u}_{Hi}& {u}_{ERi}\end{array}\right]}^{T}\sim N(0,{{\varvec{\Sigma}}}_{\mathbf{b}})$$, ${{\varvec{\Sigma}}}_{\mathbf{b}}$ being a 2 × 2 covariance matrix describing association between the mouse-specific intercepts, $${\left[\begin{array}{cc}{\epsilon }_{Hi}& {\epsilon }_{ERi}\end{array}\right]}^{T}\sim N(0,{{\varvec{\Sigma}}}_{\mathbf{e}})$$, and ${{\varvec{\Sigma}}}_{\mathbf{e}}$ being a 2 × 2 covariance matrix describing residuals and the association in residuals between traits. We used the default priors for all parameters. Since the response of the light–dark change to drug treatment will be scale-dependent, with HB magnitude and ELM-RPE thickness having different scales, we examined a normalized drug response for each trait, ${{\tilde{Y}_{{drug}} - \tilde{Y}_{{control}} } \mathord{\left/ {\vphantom {{\tilde{Y}_{{drug}} - \tilde{Y}_{{control}} } {\tilde{Y}_{{control}} }}} \right. \kern-\nulldelimiterspace} {\tilde{Y}_{{control}} }}$, where $\tilde{Y }$ represents the current estimate of the mean light–dark difference in the simulations. Comparisons of the normalized drug response were made based on simulated differences in this normalized drug response for both comparisons of DNP dose and of HB magnitude with ELM-RPE thickness.

We used hierarchical models for both F-actin and ERG responses. All regression coefficients had diffuse normal priors, and all variances had diffuse Cauchy distributions. The random intercept in each model had a normal hyperprior. The F-actin model included group (C57 + saline, C57, 129S6, and DNP-treated C57), side (apical vs basal), the interaction of group and side, and a random intercept for mouse nested within-group.

We modeled the ERG responses based on a relationship between the amplitude and log(intensity) for each wave separately. While we did consider using a non-linear model, the small number of intensities examined limited our ability to reliably fit such models. As such, we considered linear, quadratic, and cubic terms in each model. Each model also included drug treatment (DNP vs control) and the interactions between log(intensity) and drug treatment. We used leave-one-out cross-validation and WAIC^64^ to select the terms of log(intensity) to include in the final model. WAIC was slightly smaller (difference < 4) for the model that included cubic terms for both the a- and c-waves. As such, we decided to use a model that only included linear and quadratic terms, given the small improvement in fit by including cubic terms. The model with linear, quadratic, and cubic terms had the smallest WAIC for b-wave.

## Supplementary Information

Supplementary Figures.

## Data Availability

All data generated or analyzed during this study are included in this published article (and its Supplementary Information files).
