# Author Correction: Functional regulation of an outer retina hyporeflective band on optical coherence tomography images

**DOI:** 10.1038/s41598-022-12279-1

**Published:** 2022-05-16

**Authors:** Shasha Gao, Yichao Li, David Bissig, Ethan D. Cohen, Robert H. Podolsky, Karen Lins Childers, Gregory Vernon, Sonia Chen, Bruce A. Berkowitz, Haohua Qian

**Affiliations:** 1 Department of Ophthalmology, the First Affiliated Hospital, Zhengzhou University, Zhengzhou, China; 2 Visual Function Core, National Eye Institute, National Institutes of Health, Bethesda, MD 20892 USA; 3 Department of Neurology, University of California Davis, Sacramento, CA USA; 4 Division of Biomedical Physics, Office of Science and Engineering Labs, Center for Devices and Radiological Health, Food and Drug Administration, Silver Spring, MD USA; 5 Beaumont Research Institute, Beaumont Health, Royal Oak, MI 48073 USA; 6 Department of Ophthalmology, Visual and Anatomical Sciences, Wayne State University School of Medicine, Detroit, MI USA

Correction to: *Scientific reports* 10.1038/s41598-021-89599-1, published online 13 May 2021

The original version of this Article contained an error in Figure 1C, where the label “Hyporeflective Band” was mislabelled as “Hyperreflective Band”.

This error has been corrected in the original Article.

**Figure 1:**
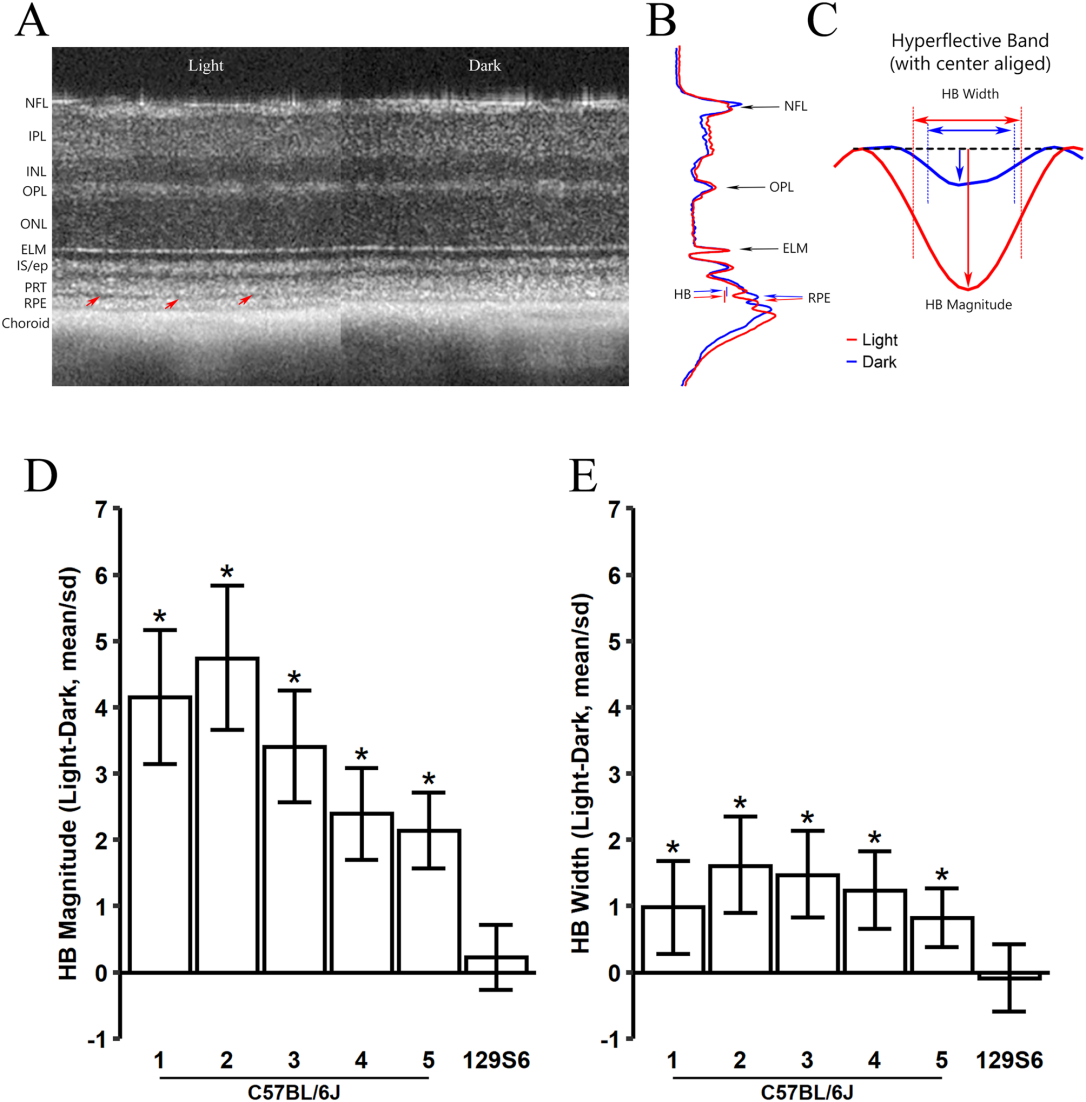
Effect of light and dark adaptation on the HB in outer retina. (**A**) Example of OCT images captured in light and dark from the same mouse eye. Red arrows point the HB between photoreceptor tip (PRT) and retinal pigment epithelium (RPE) layers. *NFL* nerve fiber layer, *IPL* inner plexiform layer, *INL* inner nuclear layer, *OPL* outer plexiform layer, *ONL* outer nuclear layer, *ELM* external limiting membrane, *IS/ep* inner segment/ellipsoid layer. (**B**) Representative intensity profiles averaged from all four radial scans of the eye in (**A**) for images captured in light (red) and dark (blue). HB is marked by a bar on left side. (**C**) Magnified view of HB. The dashed line represents the baseline constructed by connecting the maximal intensity for photoreceptor tip layer and RPE layer (Supplemental Figure S2) and rotated to horizontal. The intensity profiles are shifted laterally with center aligned for light and dark conditions. HB magnitude is calculated as the peak distance from the baseline line; HB width is measured as length between two half maximal points. Normalized light–dark responses of HB magnitude (**D**) and width (**E**) were made from 5 C57BL/6J mouse groups (Table 1) and one 129S6/ev group under light and dark conditions. Values are normalized by standard deviation of each mouse group. An asterisk indicates the credibility interval excludes 0 (equivalent to statistically significant).

