# Supplementary material for: Functional regulation of an outer retina hyporeflective band on optical coherence tomography images
Source: Sci Rep. 2021 May 13;11:10260. doi: 10.1038/s41598-021-89599-1 (PMC8119672; doi:10.1038/s41598-021-89599-1)
Supplement: Supplementary file 1 — Supplementary Figures. [file 41598_2021_89599_MOESM1_ESM.pdf]

Functional regulation of an outer retina hyporeflective band on optical coherence tomography images

Shasha Gao[1,2], Yichao Li[2], David Bissig[3], Ethan D. Cohen[4], Robert H. Podolsky[5], Karen Lins Childers[5], Gregory Vernon[2], Sonia Chen[2], Bruce A. Berkowitz[6] and Haohua Qian[2*]

[1]Department of Ophthalmology, the First Affiliated Hospital of Zhengzhou University, Zhengzhou, China; [2]Visual Function Core, National Eye Institute, National Institutes of Health, Bethesda, MD; [3]Department of Neurology, University of California Davis, Sacramento, CA; [4]Division of Biomedical Physics, Office of Science and Engineering Labs, Center for Devices and Radiological Health, Food and Drug Administration, Silver Spring, MD; [5]Beaumont Research Institute, Beaumont Health, Royal Oak, MI 48073; [6]Department of Ophthalmology, Visual and Anatomical Sciences, Wayne State University School of Medicine, Detroit, MI

*Corresponding author: Haohua Qian, PhD
Visual Function Core
National Eye Institute
National Institutes of Health
Bethesda, MD 20892

Phone: (301) 435-6275
Fax: (301) 451-5785
e-mail: Haohua.qian@nih.gov

Supplementary Information

Figure S1:

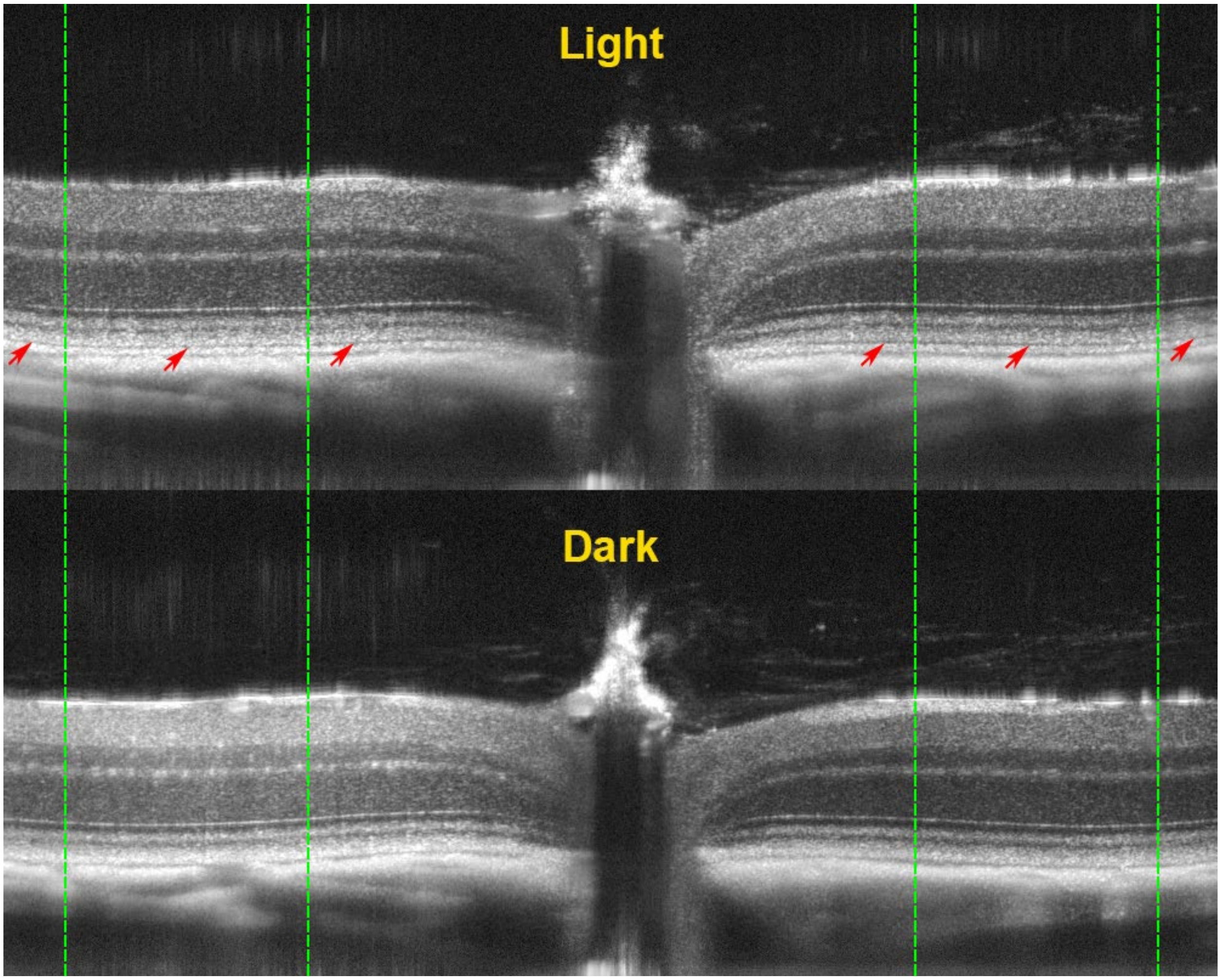

Figure S1: Example of full-frame OCT images captured in light and dark from the same mouse eye. Green dash lines delineate regions of OCT images used for analysis (i.e. 51-200 pixels from each end). Red arrows point to HB between photoreceptor tip layer and RPE layer.

Figure S2:

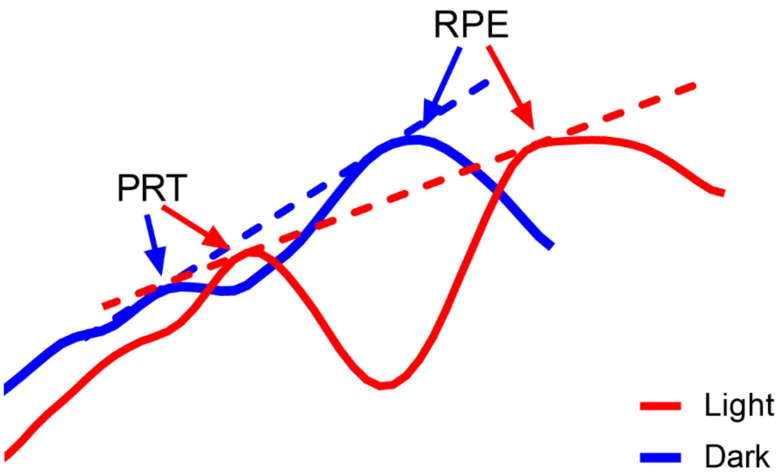

Figure S2: OCT intensity in HB region under light- and dark-adapted conditions. HB region of OCT intensity profiles shown in Figure 1B for images captured in light (Red) and dark (Blue). The dashed line represents the baseline constructed by connecting the maximal intensity for photoreceptor tip layer and RPE layer.

Figure S3:

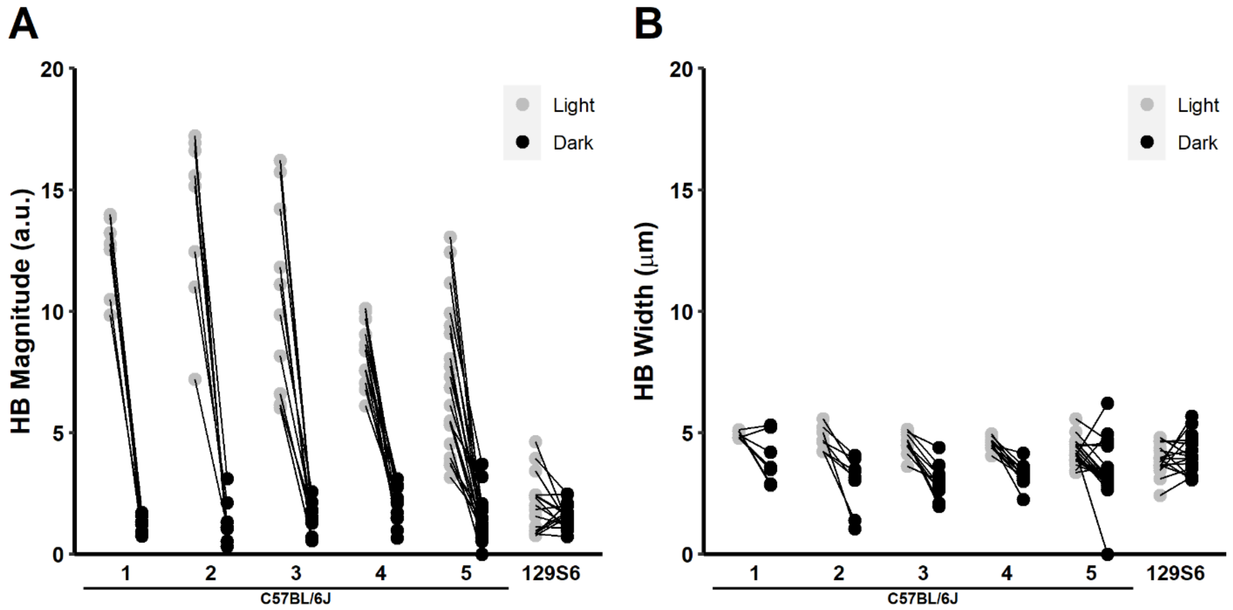

Figure S3: HB magnitude and width under light- and dark-adapted conditions. HB magnitude (A) and width (B) for six groups of mice (listed in Table 1) under light- and dark-adapted conditions. Lines connect light- and dark-adapted values for the same mouse eye.

Figure S4:

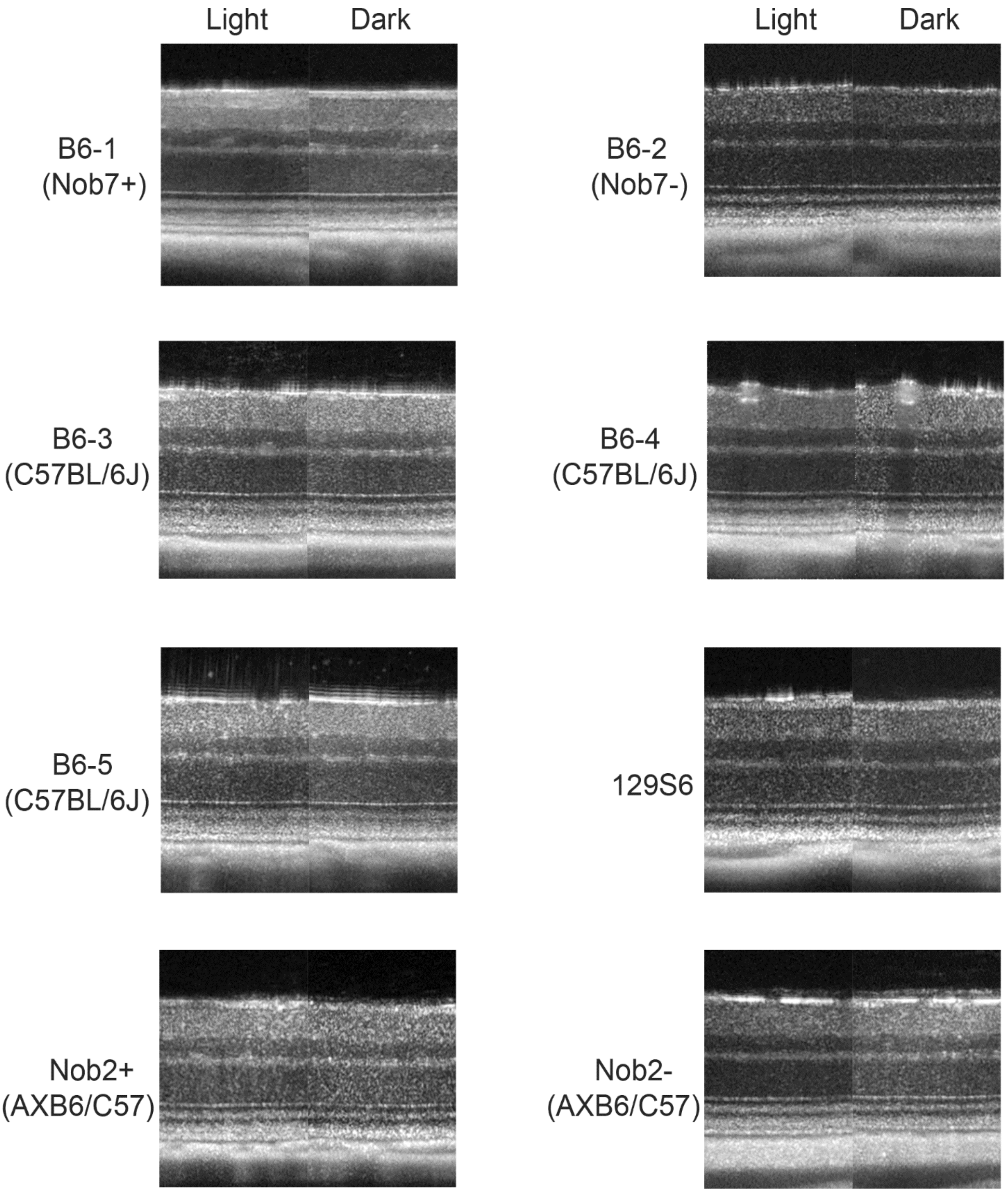

Figure S4: Examples of OCT images captured from light- and dark-adapted conditions.

Images from the same eye from eight groups of mice listed in Table 1 are illustrated.

Figure S5:

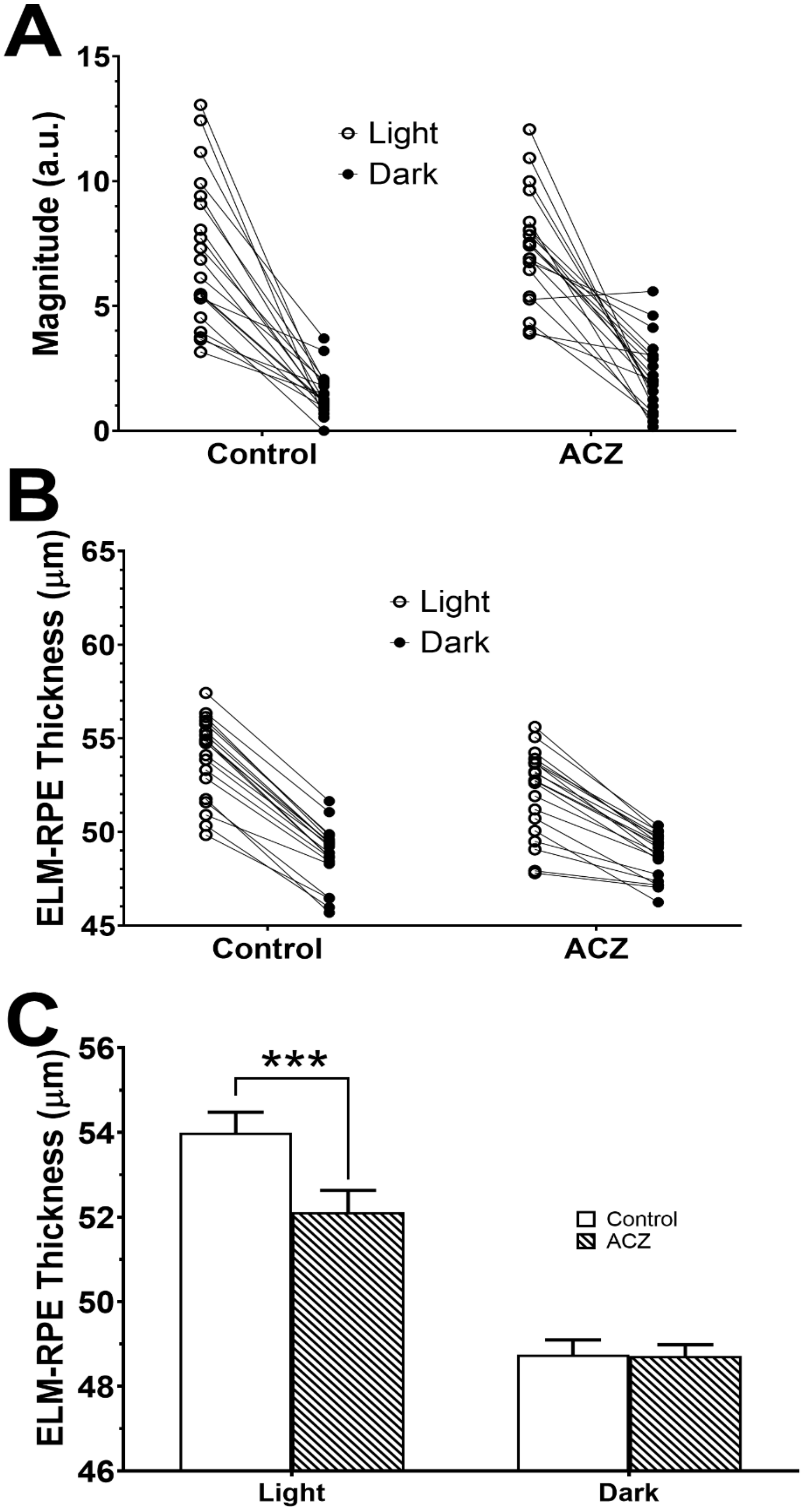

Figure S5: Effect of ACZ on outer retina responses. HB magnitude (A) and ELM-RPE thickness (B) under light- and dark-adapted conditions for control (baseline) and after ACZ-treatment. Lines connect light- and dark-adapted values for the same mouse eye. n=10 mice.

Figure S6:

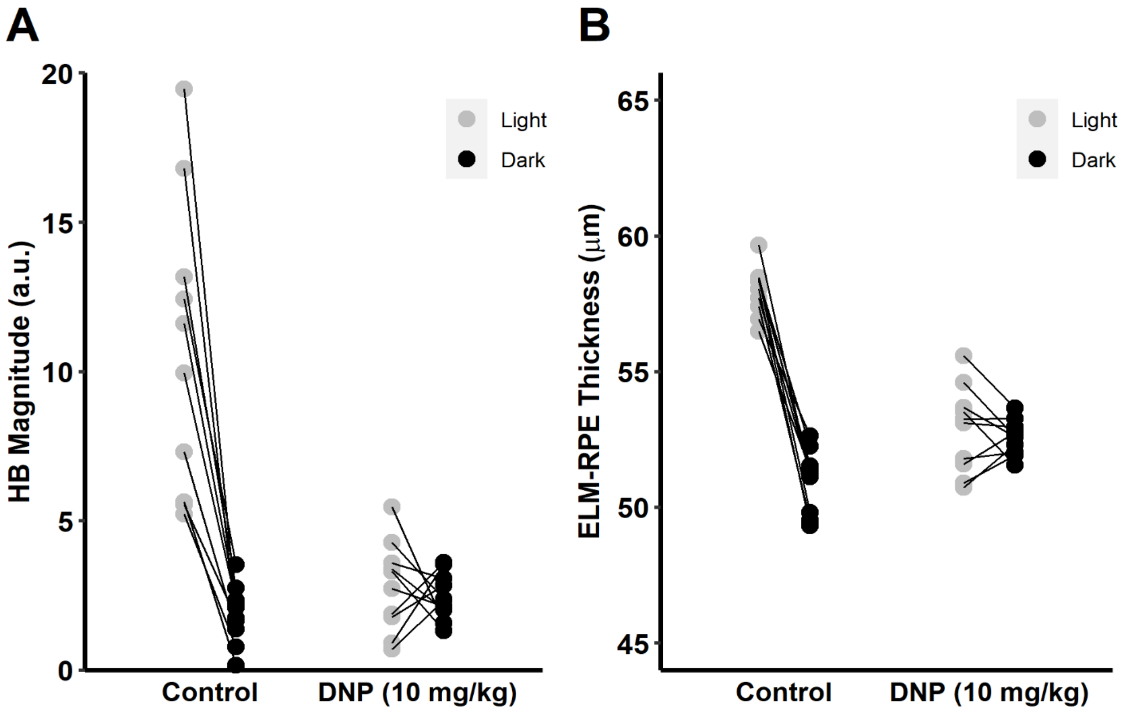

Figure S6: Effect of DNP on outer retina responses. HB magnitude (A) and ELM-RPE thickness (B) under light- and dark-adapted conditions for control (baseline) and after DNP (10 mg/kg)-treatment. Lines connect light- and dark-adapted values for the same mouse. (10 mg/kg DNP, n=5 mice; 5 mg/kg DNP, n=6 mice).

Figure S7:

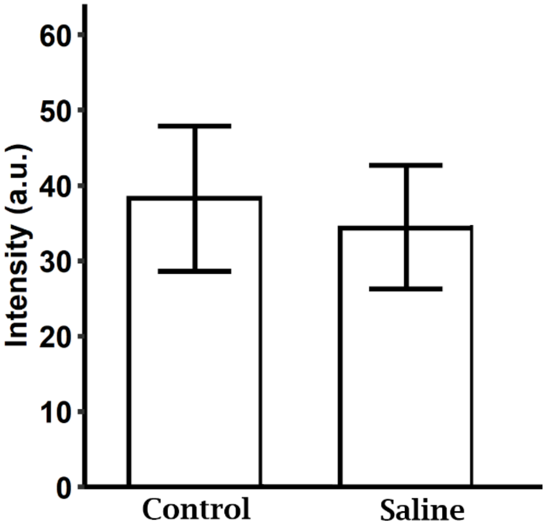

Figure S7: Saline injection does not affect F-actin distribution. Mean phalloidin fluorescent intensity measured at HB regions. All error bars represent 95% credibility intervals. (Control, n=6; Saline, n=8).

Figure S8:

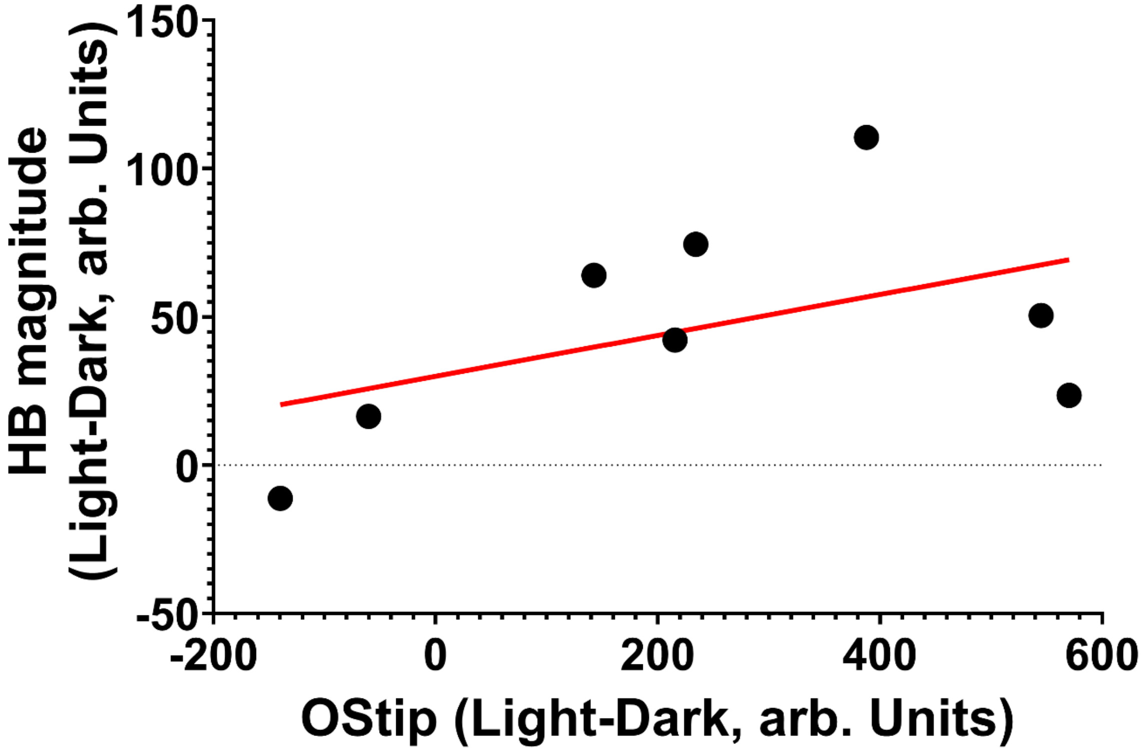

Figure S8: Correlation of Light-dark HB magnitude changes with OCT intensity of PRT (OStip) band in human subjects. PRT intensities were measured from aligned intensity profile in the dataset. No significant correlation (p=0.24) among the data, with correlation coefficient of 0.48 and 95% confidence interval of -0.35 to 0.88. Red line is regression through the data. (n=8)

Figure S9:

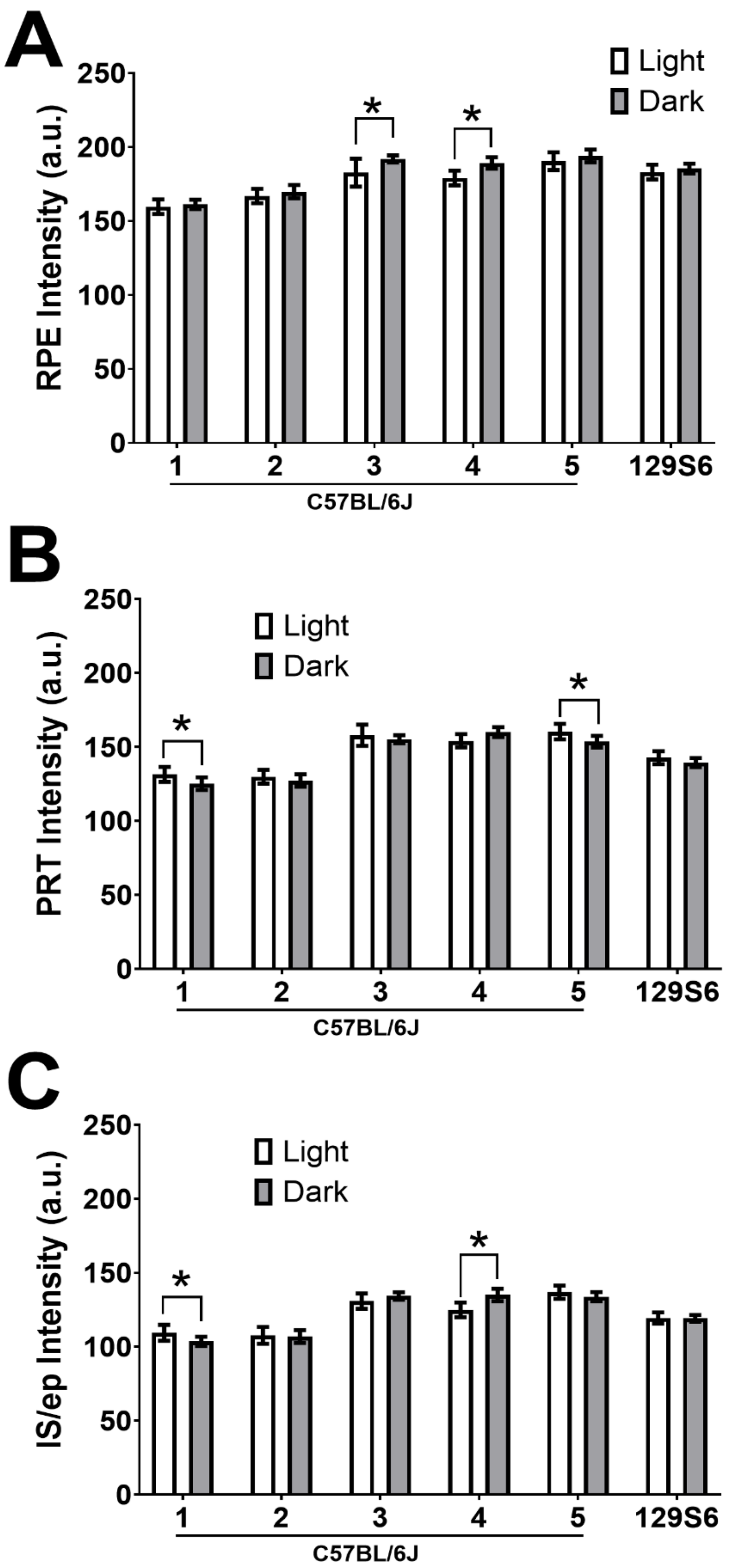
A
Light
Dark
RPE Intensity (a.u.)
250
200
150
100
50
0
*
*
1
2
3
4
5
129S6
C57BL/6J
B
Light
Dark
PRT Intensity (a.u.)
250
200
150
100
50
0
*
*
1
2
3
4
5
129S6
C57BL/6J
C
Light
Dark
IS/ep Intensity (a.u.)
250
200
150
100
50
0
*
*
1
2
3
4
5
129S6
C57BL/6J

Figure S9: OCT intensities of RPE (A), PRT(B), and IS/ep layers of mouse retina under light- and dark-adapted conditions. Band intensity were measured from averaged intensity profile. Mouse groups are listed in Table 1. There are no consistent light-dark differences in the OCT intensities for these two retinal layers. *, $p<0.05$.
